# In silico screening of protein-binding peptides with an application to developing peptide inhibitors against antibiotic resistance

**DOI:** 10.1093/pnasnexus/pgae541

**Published:** 2024-11-27

**Authors:** Xianjin Xu, Wei-Ling Kao, Allison Wang, Hsin-Jou Lee, Rui Duan, Hannah Holmes, Fabio Gallazzi, Juan Ji, Hongmin Sun, Xiao Heng, Xiaoqin Zou

**Affiliations:** Department of Physics, University of Missouri, Columbia, MO 65211, USA; Department of Biochemistry, University of Missouri, Columbia, MO 65211, USA; Dalton Cardiovascular Research Center, University of Missouri, Columbia, MO 65211, USA; Institute of Data Science and Informatics, University of Missouri, Columbia, MO 65211, USA; Department of Biochemistry, University of Missouri, Columbia, MO 65211, USA; Department of Medicine, University of Missouri, Columbia, MO 65211, USA; Department of Pharmacology, National Yang Ming Chiao Tung University College of Medicine, Taipei 112304, Taiwan; Department of Biochemistry, University of Missouri, Columbia, MO 65211, USA; Department of Medicine, University of Missouri, Columbia, MO 65211, USA; Department of Pharmacology, National Yang Ming Chiao Tung University College of Medicine, Taipei 112304, Taiwan; Department of Biochemistry, University of Missouri, Columbia, MO 65211, USA; Department of Medicine, University of Missouri, Columbia, MO 65211, USA; Department of Pharmacology, National Yang Ming Chiao Tung University College of Medicine, Taipei 112304, Taiwan; Department of Physics, University of Missouri, Columbia, MO 65211, USA; Department of Biochemistry, University of Missouri, Columbia, MO 65211, USA; Dalton Cardiovascular Research Center, University of Missouri, Columbia, MO 65211, USA; Institute of Data Science and Informatics, University of Missouri, Columbia, MO 65211, USA; Department of Biochemistry, University of Missouri, Columbia, MO 65211, USA; Molecular Interactions Core, University of Missouri, Columbia, MO 65211, USA; Department of Chemistry, University of Missouri, Columbia, MO 65211, USA; Department of Biochemistry, University of Missouri, Columbia, MO 65211, USA; Department of Medicine, University of Missouri, Columbia, MO 65211, USA; Department of Biochemistry, University of Missouri, Columbia, MO 65211, USA; Department of Physics, University of Missouri, Columbia, MO 65211, USA; Department of Biochemistry, University of Missouri, Columbia, MO 65211, USA; Dalton Cardiovascular Research Center, University of Missouri, Columbia, MO 65211, USA; Institute of Data Science and Informatics, University of Missouri, Columbia, MO 65211, USA

**Keywords:** in silico peptide screening, molecular docking, peptide drug discovery, protein–peptide interactions, antibiotic resistance

## Abstract

The field of therapeutic peptides is experiencing a surge, fueled by their advantageous features. These include predictable metabolism, enhanced safety profile, high selectivity, and reduced off-target effects compared with small-molecule drugs. Despite progress in addressing limitations associated with peptide drugs, a significant bottleneck remains: the absence of a large-scale in silico screening method for a given protein target structure. Such methods have proven invaluable in accelerating small-molecule drug discovery. The high flexibility of peptide structures and the large diversity of peptide sequences greatly hinder the development of urgently needed computational methods. Here, we report a method called MDockPeP2_VS to address these challenges. It integrates molecular docking with structural conservation between protein folding and protein–peptide binding. Briefly, we discovered that when the interfacial residues are conserved, a sequence fragment derived from a monomeric protein exhibits a high propensity to bind a target protein with a similar conformation. This valuable insight significantly reduces the search space for peptide conformations, resulting in a substantial reduction in computational time and making in silico peptide screening practical. We applied MDockPeP2_VS to develop peptide inhibitors targeting the TEM-1 β-lactamase of *Escherichia coli*, a key mechanism behind antibiotic resistance in gram-negative bacteria. Among the top 10 peptides selected from in silico screening, TF7 (KTYLAQAAATG) showed significant inhibition of β-lactamase activity with a *K*_i_ value of 1.37 ± 0.37 µM. This fully automated, large-scale structure-based in silico peptide screening software is available for free download at https://zougrouptoolkit.missouri.edu/mdockpep2_vs/download.html.

**Significance Statement**

To address the unmet need for mechanistic studies in signal transduction pathways and peptide lead discovery for therapeutic intervention and probe design, we developed MDockPeP2_VS, a systematic and large-scale structure-based in silico peptide screening method. This approach overcomes the challenges of peptide flexibility and sequence diversity. It integrates molecular docking with structural conservation between protein folding and protein–peptide binding. MDockPeP2_VS enables the fully automated design of peptides targeting *Escherichia coli* β-lactamase, a key factor in antibiotic resistance, and achieves a significant inhibition with a *K*_i_ value of 1.37 ± 0.37 µM. This tool serves as a fully automated in silico screening method for discovering unreported protein-binding peptides and is applicable to any target protein with an available atomic structure.

## Introduction

Peptides, short chains of amino acids typically containing fewer than 40 residues, have garnered significant interest as potential drug candidates within the pharmaceutical industry. In recent years, peptides have experienced notable success due to several advantageous features, including their predictable metabolism, enhanced safety profile, high selectivity, and low off-target effects when compared with small-molecule drugs (1–3). Over 30 noninsulin peptide drugs have been approved since 2000, with >170 peptides currently in active clinical development (1). While progress has been made in addressing the inherent weaknesses of peptide drugs, particularly their pharmacokinetic properties (2), one persistent challenge in peptide drug discovery remains: the development of large-scale in silico screening methods, which have proven invaluable in accelerating small-molecule drug discovery (4–6). Surprisingly, to the best of our knowledge, no reported large-scale in silico peptide screening method exists that can efficiently search for lead peptides targeting any protein.

Early efforts in peptide lead discovery were limited to naturally occurring peptides (such as human hormones) and natural product–derived peptides (such as venoms and toxins) (7, 8). However, such peptides are not available for many disease targets. In the case of protein–protein interactions (PPIs), it is usually difficult to find a PPI fragment as a starting lead if the PPI involves amino acids close in space but distal in sequence. Library-based methods, such as phage, ribosome, and mRNA display (9–11), and high-throughput screening of synthetic peptide libraries (12) achieved great successes in the discovery of de novo peptide leads. In addition to the disadvantages of most experimental methods (e.g. costly and time-consuming), a practical limitation of these library-based methods is the size of peptide libraries. Considering a 10-mer peptide where each position may be occupied by one of the 20 canonical amino acids, the resultant library of ∼10^13^ sequences is significantly larger than the library diversity of the widely used phage display methods (∼10^9^) and is close to the upper limitation (∼10^14^) of mRNA display libraries (13). Therefore, current experimental library-based methods can only reach the whole sequence space for short peptides (normally ≤10-mer) and a fraction of possible sequences for medium-size and long peptides.

On the other hand, the development of in silico peptide screening methods is far behind the aforementioned experimental methods. A major reason is that peptides are highly flexible, resulting in a huge number of degrees of freedom in the binding mode sampling process, making it challenging to efficiently predict binding modes/affinities of protein–peptide complexes, especially for cases containing medium-size or long peptides (≥10-mer). To date, only a few successful cases have been reported on the de novo sequence design of protein-binding peptides or miniproteins. For example, recent studies by David Baker and coworkers successfully designed peptides with constrained stable conformations (14), which can be used as starting scaffolds for peptide library preparation. They also successfully designed de novo miniprotein binders (50–65 amino acids) for diverse protein targets using a strategy combining both computational and experimental methods (15). Other remarkable computational efforts were devoted to optimizing or re-designing sequences to achieve more potent, selective, or stable protein-binding peptides than their known parent peptides (16–19). Although many protein–peptide docking methods have recently been developed, unlike the molecular docking methods for in silico small-molecule screening, the existing peptide docking methods are impractical for large-scale in silico peptide screening mainly due to their high computational demand (20).

**Fig. 1.**
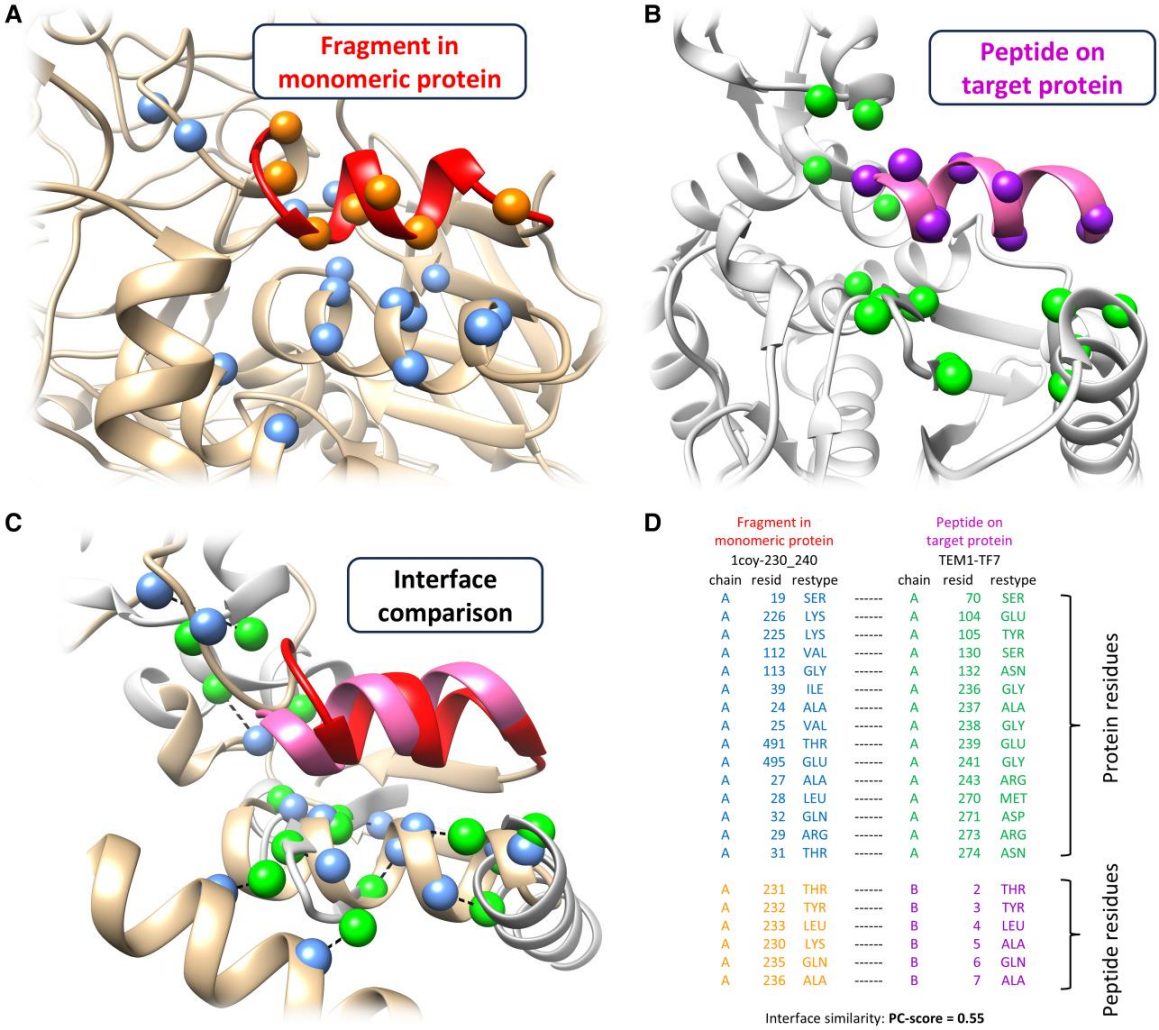
Illustration of the key idea of MDockPeP2_VS. A fragment derived from a monomeric protein (A) is highly likely to bind to a target protein (B) with a similar conformation when the interfacial residues are conserved (C and D). A) A fragment (residues 230–240, colored red) from a monomeric protein (PDB ID: 1coy). B) The binding mode of the peptide TF7 (highlighted in hot pink) on the target protein (TEM-1; PDB ID: 1s0w, chain A) predicted by the docking engine implemented in MDockPeP2_VS. TF7 shares the identical sequence with the red fragment displayed in (A). C and D) The two interfaces (A and B) are superimposed, and their physicochemical similarity is measured by the program PCalign (22). A PC score of 0 indicates no similarity, while a PC score of 1 reflects complete identity. The Cα atoms of conserved residues at the interface are shown as blue and green spheres in (A–C), respectively. Dashed lines in (C) indicate the correspondence between each conserved residue pair’s Cα atoms.

In this study, we introduced a systematic, large-scale structure-based in silico peptide screening method, referred to as MDockPeP2_VS. The idea behind MDockPeP2_VS was inspired by the conservation between peptide–protein binding and protein folding. Our previous study revealed that peptides on protein surfaces and fragments in monomeric proteins tend to form similar conformations when peptides and fragments share similar sequences and similar interacting interfaces (21). Therefore, we assume that a sequence fragment extracted from a monomeric protein structure is likely to bind a target protein if the predicted binding mode of the fragment on the target protein retains most of the physicochemical interactions of the same fragment observed in the monomeric protein (see Fig. 1). This assumption can dramatically reduce the search space for peptide conformations, making the computational time affordable. The details of MDockPeP2_VS are described in the Materials and methods section.

As a proof-of-concept study, MDockPeP2_VS was applied to the development of peptide inhibitors targeting the TEM-1 β-lactamase of *Escherichia coli* (23), which is one of the most prominent mechanisms of antibiotic resistance in gram-negative bacteria (24). Antibiotic resistance is one of the greatest public health threats worldwide. β-Lactam antibiotics are the major class of antibiotics that contain a β-lactam ring moiety, which can be hydrolyzed by β-lactamases in antibiotic-resistant bacteria (24). Small-molecule β-lactamase inhibitors, such as clavulanate, have been developed and co-administered with β-lactam antibiotics such as amoxicillin to overcome resistance. Augmentin (amoxicillin/clavulanate) is considered an essential medicine by the World Health Organization and has been widely used to treat various bacterial infections (25). However, resistance to small-molecule inhibitors has developed due to bacterial mutations, so there is an urgent clinical need for new β-lactamase inhibitors (26). Therefore, TEM-1 β-lactamase is an ideal target for validating MDockPeP2_VS.

Briefly, we developed an automated, large-scale structure-based in silico peptide screening method, MDockPeP2_VS, which was successfully applied to develop peptide inhibitors targeting the TEM-1 β-lactamase, a key factor for antibiotic resistance. MDockPeP2_VS could be an attractive complement to valuable experimental technologies like phage display for rapid peptide screening at a much lower cost.

## Results

### Pipeline of MDockPeP2_VS

**Fig. 2.**
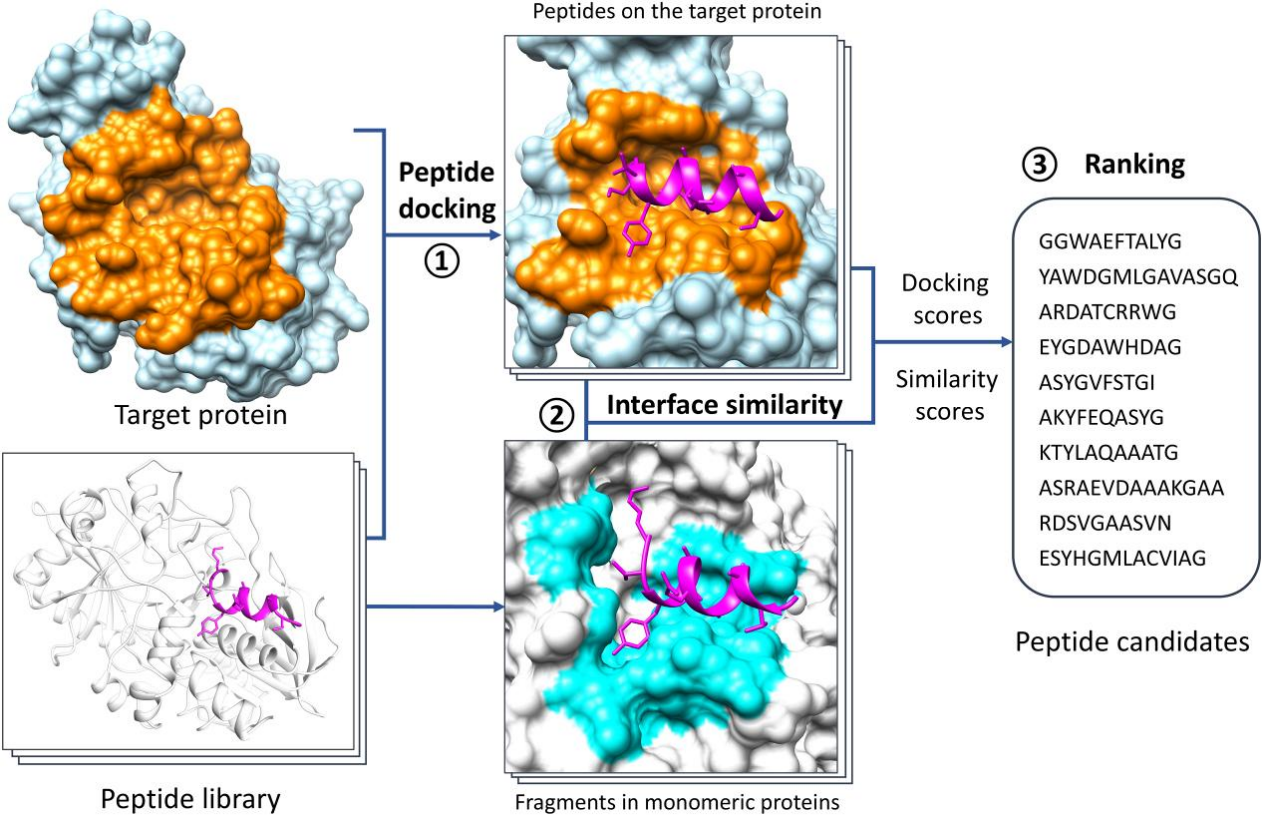
The flow chart of MDockPeP2_VS, which can be briefly divided into three steps. Step 1: protein–peptide docking. Peptides in a newly constructed peptide library are docked to a target protein using the program Vina_pep. The peptide library consists of fragments (10–15 amino acids) extracted from monomeric protein structures deposited in the PDB. Step 2: interface comparison. For each peptide, the predicted protein–peptide interacting interface is compared with the interface of the corresponding sequence fragment in the monomeric protein. Step 3: ranking. Peptides in the library are ranked by a hybrid scoring function, which combines protein–peptide docking scores and interface similarity scores. A few dozen peptide candidates are selected for synthesis and experimental validation. See the main text for details.

Figure 2 shows the flow chart of MDockPeP2_VS. Initially, a new peptide library was constructed by extracting fragments from experimentally determined monomeric protein structures in the Protein Data Bank (PDB) (27). The screening library used in the study consists of 76,223 nonredundant peptides with sequence lengths between 10 and 15 amino acids, and is available for free download at https://zougrouptoolkit.missouri.edu/mdockpep2_vs/download.html. Notably, these fragments form alpha helices in the monomeric proteins. We assume that candidate peptides would also form helical structures on the target protein TEM-1 when the interfacial residues are conserved (also see Fig. 1).

Based on the above assumption, we treated the peptide backbone conformation as rigid during docking, which significantly reduced the search space for peptide conformations. This in turn decreased computational time and improved cost-effectiveness. The peptide side chains remained fully flexible during the docking process.

After the docking processes, peptides in the library were ranked using our hybrid scoring function called PepProScore (21), which combines the protein–peptide-binding score (Vina_Score) (28) and the interface conservation score (PC_Score) (22). The PC_Score of a peptide was calculated by comparing the predicted protein–peptide interacting interface with the corresponding fragment in the monomeric protein.

Finally, a few dozen peptide candidates were selected from the ranking list for synthesis and experimental validation. To further increase the hit rate in the final ranking list, we established several filtering criteria, such as a threshold for the interface conservation score, the amino acid composition of peptides, and consensus of modeled protein–peptide complex structures with other computational methods.

Details of each step in MDockPeP2_VS are available in the Materials and methods section.

### Docking engine Vina_pep vs. Vina

In MDockPeP2_VS, a modified version of AutoDock Vina (28), named Vina_pep, was used as the docking engine for the protein–peptide-binding mode prediction. AutoDock Vina is a widely used molecular docking program for predicting protein-small-molecule-binding modes. Despite its numerous successes, AutoDock Vina cannot be directly applied to protein–peptide-binding mode prediction, because it lacks the necessary algorithms and parameters for modeling peptide backbone conformations. However, this was not a concern for our peptide screening strategy, in which a peptide conformer was pregenerated for each entry in the peptide library and the backbone conformation was treated as being rigid during docking processes. A major concern of applying AutoDock Vina to peptide screening was the computational speed, especially for medium-sized and long peptides. Testing of AutoDock Vina (default settings with *exhaustiveness* value 8) on the benchmarking PepPro dataset (29), which consists of 89 nonredundant protein–peptide complexes with peptide sequence lengths ranging from 5 to 29, showed an average of 3.6 CPU hours for each docking run on an Intel(R) Xeon(R) Processor E5-1650 v3 (3.50 GHz). The demanding computational cost of AutoDock Vina on protein–peptide docking limits its application to screening large-scale peptide libraries. Remarkably, Vina_pep (default settings with *exhaustiveness* value 64) took an average of 13 min per core for one protein–peptide docking, which was about 16 times faster than AutoDock Vina.

In addition to the computational cost, we further evaluated the performance of Vina_pep and Vina in predicting protein–peptide-binding modes based on the PepPro dataset. Docking modes of each complex were compared with the experimentally determined complex structure using a criterion referred to as the critical ligand root-mean-square deviation (cL-RMSD) (21). Specifically, the protein structures were superimposed, and the RMSD was calculated for the heavy atoms of all the peptide contact residues and the backbone atoms of the peptide noncontact residues. A peptide residue was identified as a contact residue if its relative buried surface area was >33.3%. The buried surface area was calculated based on the crystal complex structure using the program Naccess V2.1.1 (30).

For AutoDock Vina, when considering the top 1, 5, or 10 models for each case, the mean cL-RMSD values for the 89 peptides in the PepPro dataset were 1.8, 1.5, and 1.5 Å, respectively. The median values for the same cases were 3.0, 2.5, and 2.3 Å, respectively. Slightly lower cL-RMSD values, indicating a slightly better performance, were achieved with Vina_pep. When considering the top 1, 5, or 10 models for each case, the mean cL-RMSD values were 1.5, 1.2, and 1.1 Å, respectively. The corresponding median values were 2.1, 1.9, and 1.8 Å.

In summary, Vina_pep proved to be ∼16 times faster than its original version while maintaining accuracy in predicting protein–peptide-binding modes. As a result, Vina_pep was selected as the docking engine for our in silico screening method, MDockPeP2_VS.

### In silico screening results for the target protein TEM-1

**Fig. 3.**
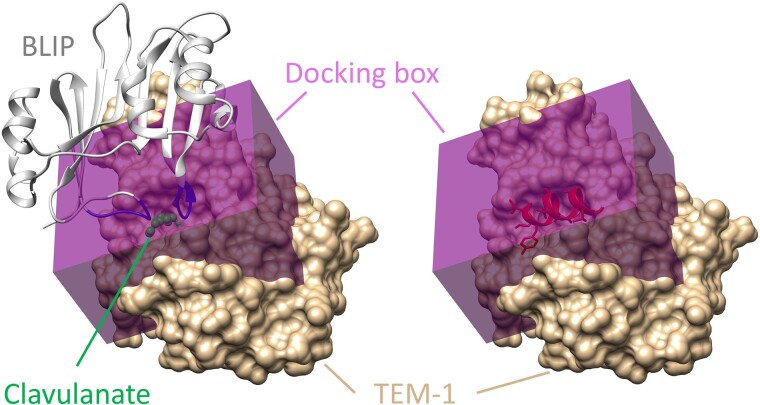
The predefined binding site on β-lactamase TEM-1. TEM-1 is represented in the surface model, colored tan (PDB ID: 1s0w, chain A). As shown in the left panel, the docking box is shaded purple and encompasses the binding site of a small-molecule drug, clavulanate (displayed in the stick and ball model and colored green), and a portion of the binding site of the BLIP (shown in the ribbon model and colored gray). The two loops of BLIP binding in the TEM-1 active site are highlighted in blue. The right panel shows an example of a screened peptide bound to TEM-1.

In this study, MDockPeP2_VS was used to discover peptide inhibitors for the β-lactamase TEM-1. Figure 3 shows the predefined peptide-binding site (docking box) on TEM-1 (PDB ID: 1s0w, chain A) (23). For comparison, a small-molecule β-lactamase inhibitor, clavulanate (31), is displayed. The binding mode of clavulanate on TEM-1 was constructed based on the structure of clavulanate bound to a homologous protein, β-lactamase from *Mycobacterium tuberculosis* (PDB ID: 3cg5) (32), which shares 54% sequence similarity with TEM-1. Figure 3 also shows the binding location of the β-lactamase inhibitor protein (BLIP, PDB ID: 1s0w, chain B) (23), which was produced from *Streptomyces clavuligerus*. In the peptide screening studies, the docking box was set to 36 Å × 30 Å × 20 Å with the geometric center at (36.9, 25.0, 48.1 Å), which included the whole binding site of clavulanate and part of the binding site of BLIP.

The in silico screening was performed on a high-performance computing (HPC) cluster supported by the University of Missouri Bioinformatics Consortium (UMBC). A protein–peptide docking took an average of 5.3 (±3.2) min using a single core of an Intel(R) Xeon(R) CPU E5-2680 v4 (A 2.40 GHz). The screening was completed in 8 h using 1,000 compute cores.

The top 10 peptide candidate outputs from MDockPeP2_VS are reported in Table 1. The sequence lengths of candidate peptides range from 10 to 14. Their sequence locations in original monomeric proteins are also listed in the table. Here, we focused on the peptides with PC_scores (interface similarity score) >0.5, which were subsequently synthesized for the β-lactamase activity assay.

**Table 1.** The 10 peptide candidates selected from the MDockPeP2_VS screening for TEM-1.

Peptide ID	Sequence	Length	Fragments in PDB^a^	PepProScore	PC_Score	L-RMSD^b^ (Å)
TF1^c^	GGWAEFTALYG	11	4cimA_142-152	−12.95	0.55	9.98
TF2^c^	YAWDGMLGAVASGQ	14	4dz1A_75-88	−12.89	0.55	5.61
TF3	ARDATCRRWG	10	3ofkA_148-157	−12.71	0.50	9.0
TF4	EYGDAWHDAG	10	1yr2A_530-539	−12.41	0.52	5.86
TF5^c^	ASYGVFSTGI	10	2ocaA_403-412	−12.40	0.50	5.16
TF6	AKYFEQASYG	10	2be9A_276-285	−12.29	0.53	11.08
TF7	KTYLAQAAATG	11	1coyA_230-240	−12.12	0.55	3.1
TF8	ASRAEVDAAAKGAA	14	4mrsA_462-475	−12.11	0.50	14.37
TF9	RDSVGAASVN	10	4bocA_936-945	−12.09	0.54	5.85
TF10^c^	ESYHGMLACVIAG	13	5tpiA_216-228	−12.08	0.59	4.8

^a^The corresponding sequence fragment in a monomeric protein, written in the following format: PDB ID, chain ID, and the sequence numbers of the start and end residues.

^b^Comparison of protein–peptide-binding modes predicted by AlphaFold-Multimer and MDockPeP2_VS.

^c^Peptides that have low solubility were excluded from assay experiments.

### Experimental validation results

**Fig. 4.**
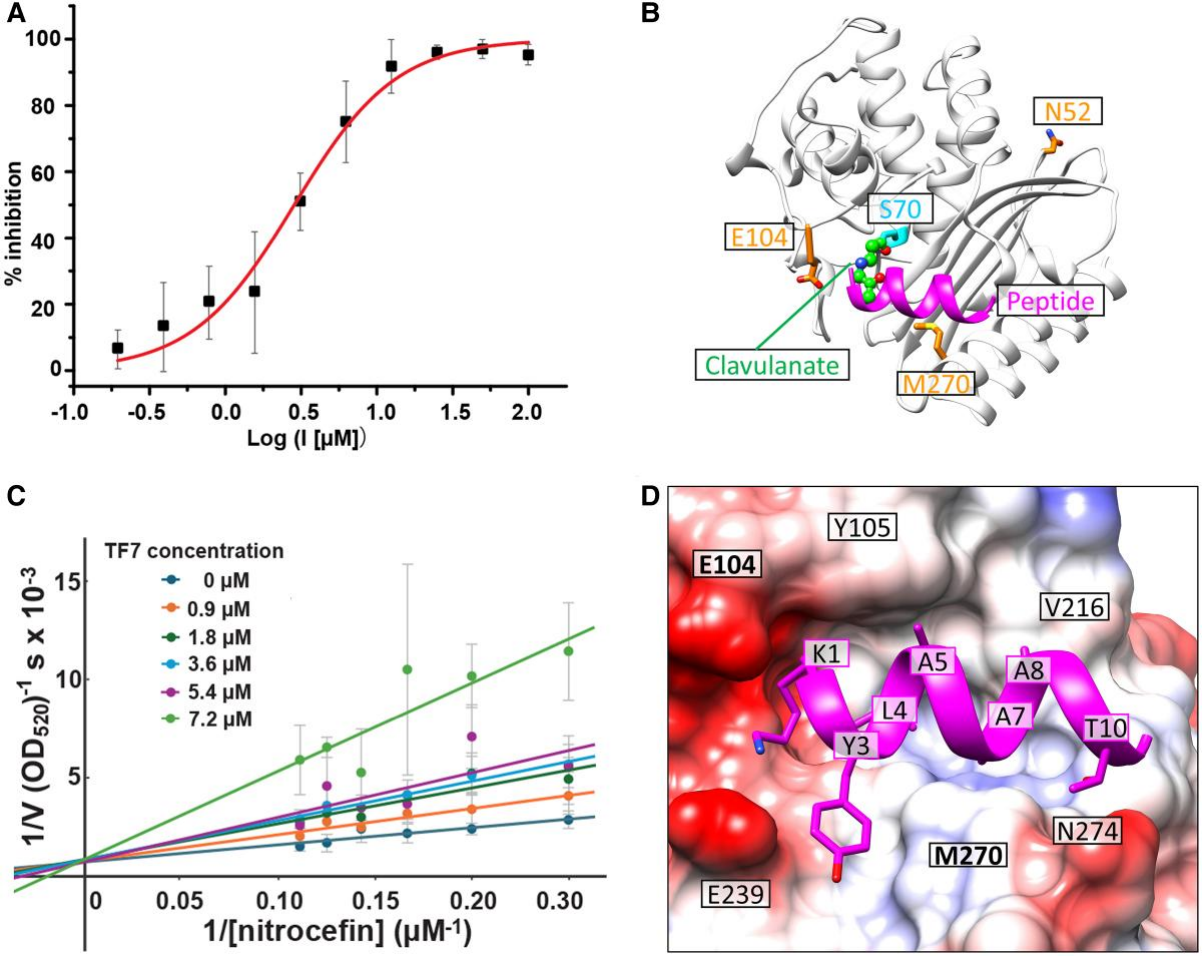
Experimental and modeling results of the peptide TF7 for the target protein, TEM-1. A) Inhibition curve of TF7 for the β-lactamase activity of TEM-1. B) The location of TF7 (colored magenta) bound to TEM-1. For comparison, the β-lactamase inhibitor, clavulanate (displayed in the stick and ball representation), is also displayed. Clavulanate is covalently attached to S70 of TEM-1. C) Lineweaver–Burk double reciprocal plot analysis depicting the TEM-1 inhibition kinetics by peptide TF7. A competitive inhibition mechanism was observed, as the 1/*V*_max_ (Y-intercept) was unaffected under various peptide concentrations. D and E) Details of the interaction between TF7 and TEM-1. In (D), the peptide sidechains are shown in stick representation. TEM-1 is shown in surface representation with Coulombic surface coloring. Red regions are overall negatively charged, blue regions are overall positively charged, and white regions are hydrophobic. Figures presenting protein/peptide structures were prepared with UCSF Chimera (33).

Top 10 ranked peptides were synthesized and purified. Four of these 10 peptides have low solubility (see Table 1), and we therefore focused on the remaining 6 peptides. Because these peptides were designed to bind to the substrate-binding pocket of TEM-1, the interactions of TEM-1:peptide were evaluated using β-lactamase activity assay. The peptides were preincubated with purified recombinant TEM-1 at room temperature to allow binding before mixing with substrate, nitrocefin. The enzymatic activity was measured by monitoring the colorimetric change of nitrocefin with TEM-1 in the absence and presence of the peptide candidates. Three of the 6 soluble peptides changed the β-lactamase activity of TEM-1 at the peptide screening concentration of 100 µM. TF7 (KTYLAQAAATG, fragment 230–240 from PDB ID: 1coy, chain A) showed significant inhibition of the β-lactamase activity, while TF3 and TF6 increased the β-lactamase activity of TEM-1. Dose–response inhibition analysis was then performed on TF7, and its inhibition constant (*K*_i_) was calculated as 1.37 ± 0.37 µM (Fig. 4A). It is approximately two orders of magnitude more potent than the reported TEM-1 β-lactamase peptide inhibitor, RRGHYY-NH2 (*K*_i_ = 136 μM), which was discovered by phage display (34).

Clavulanate, a Food and Drug Administration–approved small molecule, effectively inhibits the β-lactamase activity of TEM-1 by forming a covalent bond with S70 of TEM-1 upon hydrolysis of the β-lactam ring (31). Superimposing the clavulanate-bound TEM-1 structure and the TF7-bound TEM-1 structure suggests that our peptide targets the same drug-binding pocket (Fig. 4B). Lineweaver–Burk plots confirm that TF7 competitively inhibits TEM-1, as evidenced by the convergence of Y-intercepts across various peptide concentrations (Fig. 4C). These findings align with our predicted model that TF7 binds to the substrate binding pocket and thus inhibits TEM-1 activity.

In experimental validation, we noticed that a 30-min incubation is necessary to observe the peptides’ inhibitory effect on TEM-1, as very weak inhibitory effects were observed without incubation. Given that the peptide sequences in our peptide library were extracted from helical fragments in monomeric proteins (see the “Construction of the peptide library” section), these peptides may only adopt helical structures after binding to a target protein through the induced fit mechanism. This mechanism shifts the peptides from more coiled structures in solution to helical structures upon binding. Future studies could focus on optimizing the sequence of the hit peptide to stabilize the helical conformation in solution.

### Modeled complex structure of TF7 with TEM-1

Figure 4D presents the details of TF7 interacting with TEM-1, as predicted by MDockPeP2_VS. Specifically, K1 at the N-terminal of TF7 binds to the crevice formed by two negatively charged residues, E104 and E239. Y3 of TF7 binds to the pocket between E239 and M270. Two hydrophobic residues of TF7, L4, and A7, are fully embedded in the active site of TEM-1, surrounded by residues S70, S130, N132, N170, S235, A237, and R243. A5 and A8 of TF7 primarily interact with Y105 and V216, respectively. Residue T10 near the C-terminal of TF7 forms a hydrogen bond with N274 of TEM-1.

**Fig. 5.**
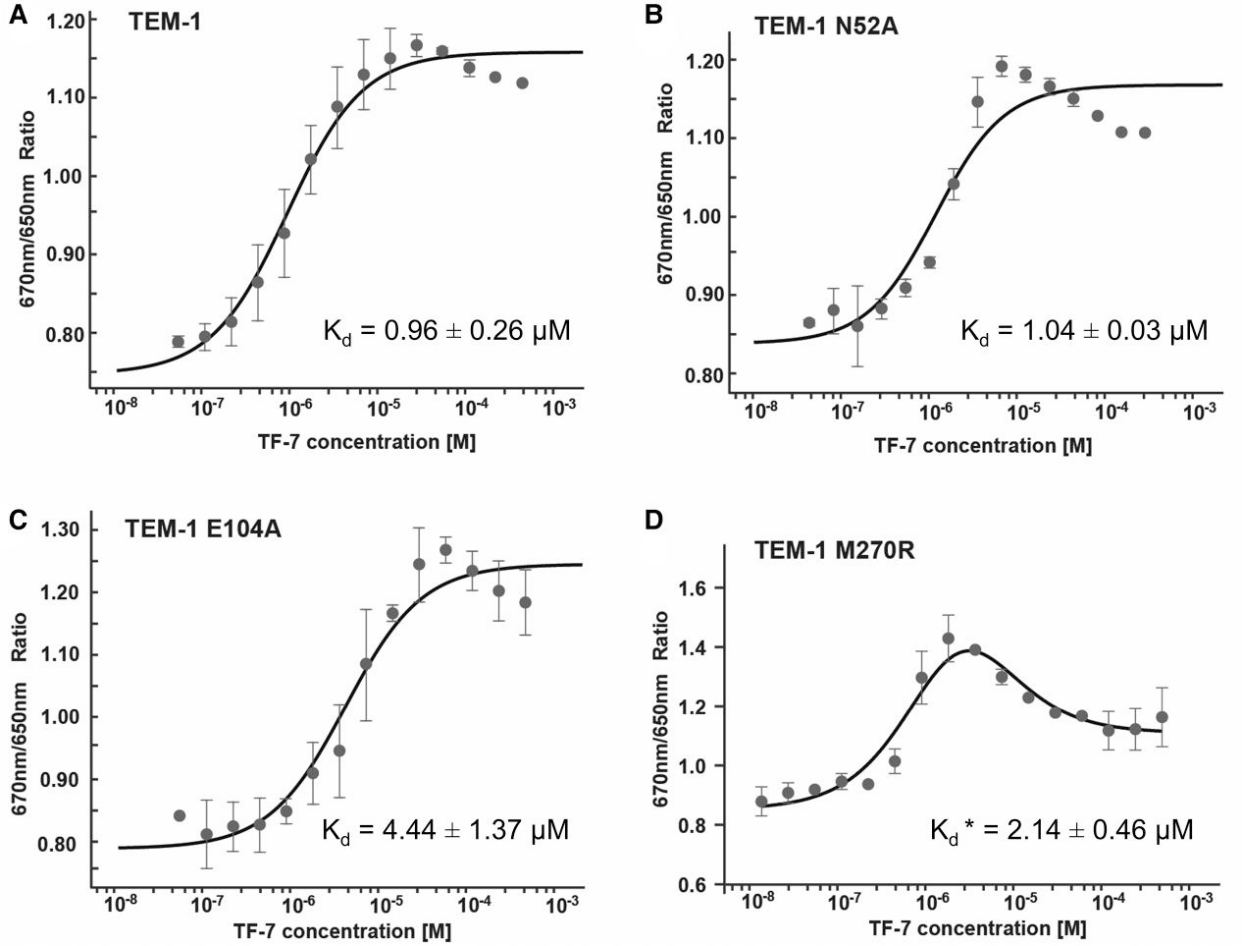
MST-binding curves of TEM-1 and mutants titrated with different concentrations of peptide TF7. A) TEM-1. B) TEM-1 N52A. C) TEM-1 E104A. D) TEM-1 M270R. The change in MST signal was fitted (black lines) to yield *K*_d_ values. The biphasic binding curve for TEM-1 M270R was analyzed as described in the Materials and methods section. Representative curves from three experiments are shown, with averages ± SD indicated. *M270R contains two TF7-binding sites. The *K*_d_ value of the site with stronger affinity is shown in (D), while the weaker affinity is 14.77 ± 12.96 µM.

To validate the predicted molecular interactions between TEM-1 and TF7, mutations were introduced to TEM-1 both within and outside of the predicted TF7 interacting sites. The binding affinities of TF7 to the wild type (WT) and mutant TEM-1 were measured by microscale thermophoresis (MST). The dissociation constant (*K*_d_) of TF7 binding to TEM-1 was 0.96 ± 0.26 µM, comparable with the measured *K*_i_. Single-point mutations E104A and M270R, designed to disrupt the TF7 interaction, exhibited weaker affinities compared with WT, as expected (Fig. 5C and D, *P* < 0.01, Student's t test). Interestingly, M270R also introduced another weaker binding site for TF7, as a biphasic binding curve was observed (Fig. 5D). The N52A mutation was designed to serve as a negative control because N52 resides on the surface of TEM-1 distal from the substrate-binding site. Indeed, its affinity to TF7 was similar to that of WT (Fig. 5B, *P* = 0.30, Student's t test). The locations of mutated residues (E104, M270, and N52) are displayed in Fig. 4B and D.

The above mutational analyses (E104A and M270R) show moderate changes (2- to 5-fold) in binding affinities (Fig. 5), indicating the advantages of peptides over small molecules in combating resistance caused by mutations: (i) peptides often have extended interaction interfaces (i.e. interaction sites) compared with small molecules (as shown in Fig. 4B), and (ii) their flexibility allows them to effectively bind to target pockets, acting like sticky patches that “glue” to the pockets. Together, these features make peptides less sensitive to point mutations in target proteins and less susceptible to resistance in bacteria.

### Consensus of binding modes modeled by MDockPeP2_VS and AlphaFold-Multimer

We further modeled the binding modes of these 10 selected peptides on TEM-1 using the AlphaFold-Multimer program (35) with default settings. This program has demonstrated promise in predicting protein–peptide interactions in recent studies (36–38). Notably, the binding modes of TF7 on TEM-1, as modeled by AlphaFold-Multimer and MDockPeP2_VS, exhibited remarkable similarity, with a L-RMSD of 3.1 Å. L-RMSD was calculated based on the backbone atoms of the peptide between the two predicted binding modes after optimal superimposition of the protein structures. The L-RMSD values for the remaining nine peptides are summarized in Table 1.

Furthermore, the binding modes of TF10, as predicted by both methods, also displayed similarity (L-RMSD = 4.8 Å). However, due to its poor solubility issue, we were unable to test its β-lactamase activity. Interestingly, L-RMSD values for the remaining peptides are >5 Å, indicating that distinct binding modes were predicted by AlphaFold-Multimer and MDockPeP2_VS. Notably, peptides with large L-RMSD values (TF3, TF4, TF6, TF8, and TF9) did not exhibit significant inhibition of the β-lactamase activity. Therefore, the consensus of protein–peptide-binding modes predicted by both MDockPeP2_VS and AlphaFold-Multimer can serve as a filtering criterion to further enhance the hit rate of our in silico screening method.

## Discussion

The peptide library used in this study consists of alpha-helical fragments with sequence lengths ranging from 10 to 15. The constructed peptide library can be significantly extended by including fragments with other types of secondary structures or fragments from modeled protein structures (e.g. from the AlphaFold protein structure database (39)), as well as short peptides (<10 amino acids) or long peptides (>15 amino acids).

Similar to the in silico screening methods for small molecules (4–6, 40), the scoring function used in our in silico peptide screening method is imperfect. In other words, MDockPeP2_VS seeks to identify an enriched subset of the potential peptide candidates for a given target protein. Applying reasonable and automated filters to the peptide ranking list can improve the hit rate. Furthermore, we observed that the consensus of the protein–peptide complex structures modeled by both MDockPeP2_VS and AlphaFold-Multimer would enhance the hit rate in peptide in silico screening. It is worth noting that AlphaFold-Multimer is computationally expensive and cannot be directly applied to large-scale peptide screening studies.

In this study, the protein structure was treated as rigid during the docking processes. Vina_pep allows sidechain flexibility of protein residues near the binding site; however, this would significantly increase the computational cost. Another way to partially consider protein flexibility is to use multiple protein conformations, which can be generated by computational methods like molecular dynamics simulations (41).

For peptides, sidechains were treated as fully flexible, and backbones were treated as rigid during dockings. As described in Fig. 1, the key idea of MDockPeP2_VS was to find peptides that share similar interactions with a target protein as the identical sequence fragments in monomeric proteins that interact with their surrounding amino acids. We assume that the backbone conformations of the candidate peptides on a target protein are close to the conformations of the corresponding sequence fragments in monomeric proteins. This assumption significantly reduces the complexity of peptide docking and enables the execution of large-scale peptide in silico screening.

In this study, TEM-1 was employed as the proof-of-concept system for our newly developed in silico peptide screening method, MDockPeP2_VS. It is noteworthy that other clinically important and emerging β-lactamases include New Delhi metallo-β-lactamase (NDM), *Klebsiella pneumoniae* carbapenemases (KPC), and CTX-M β-lactamases. The NDM β-lactamases feature an active site with two zinc atoms forming a zinc cluster. However, the current version of MDockPeP2_VS lacks parameters for ions such as zinc, rendering it unsuitable for direct application to NDM β-lactamases. On the other hand, KPC and CTX-M β-lactamases share ∼40% of sequence identity and 60% sequence similarity with TEM-1. TEM-1, KPC, and CTX-M also share similar 3D structures. Therefore, our in silico peptide screening method should also work for KPC and CTX-M types of β-lactamases, which will be the focus of our future studies.

In summary, we have developed the first large-scale, structure-based in silico peptide screening method. As a proof-of-concept study, we used the MDockPeP2_VS program based on this method to screen a peptide library constructed from helical fragments found in monomeric protein structures to target TEM-1, the β-lactamase of *E. coli* responsible for antibiotic resistance. The β-lactam-binding pocket of TEM-1 is not involved in any PPIs where the interfacial amino acids are closely positioned both in space and in sequences (as seen in the BLIP example in Fig. 3, left panel). Thus, there is no straightforward “parent template” available for designing peptide inhibitors. Our entire in silico peptide screening process was automated, and no manual examination was conducted. Out of the six peptides that were assayed, TF7 exhibited the most significant inhibition of β-lactamase activity, ∼100-fold more potent than the TEM-1 peptide inhibitor selected through phage display (34). MDockPeP2_VS is a useful tool applicable to any target protein with an available atomic structure, serving as a valuable resource for efficiently discovering peptide leads.

## Materials and methods

### Construction of the peptide library

A peptide library was constructed based on the sequence fragments in monomeric protein structures deposited in the PDB (27). In this proof-of-concept study, we focused on the fragments that form alpha helices in the monomeric proteins. First, a nonredundant protein dataset consisting of 26,517 proteins was generated based on a protein database provided by MODELLER (42) (pdb95.pir.gz, updated on 2018 June 14). The program, UCLUST (43), was employed to remove redundant proteins with a sequence similarity cutoff of 30%. Then, helical fragments were identified by using the secondary-structure information stored in their PDB files. A helical fragment together with two adjacent residues at each terminus was selected as an entry of the peptide library. A total of 76,223 peptides with sequence lengths ranging from 10 to 15 were generated in this step. The number of peptides increased to about 1.6 × 10^5^ when the maximum length of peptide sequence was set to 30. Finally, each entry in the peptide library consists of three elements: a peptide sequence, a peptide conformer, and a fragment–protein pair. The construction of the peptide conformer and the fragment–protein pair is described as follows.

The peptide conformer for each entry in the peptide library was constructed by MODELLER using the corresponding fragment structure in the monomeric protein as the template. The model refinement level was set to “*refine.fast*” to ensure that the modeled peptide conformation was close to the template structure. Sidechains that were missing in some peptides were added in the modeling process. The peptide conformers will be used to predict their binding modes with a given protein target in the in silico screening process, as described in the “in silico screening” section.

For a fragment–protein pair, which consists of the structure of a helical fragment and its surrounding protein residues, three adjacent residues at each terminus of the fragment were removed. The fragment–protein interacting interface was compared with the predicted interacting interface between the peptide and the target protein by calculating interface similarity, as illustrated in Fig. 1. This library is accessible for free download at https://zougrouptoolkit.missouri.edu/mdockpep2_vs/download.html.

### Protein–peptide docking engine

A modified version of AutoDock Vina (28), named Vina_pep, was used as the docking engine for predicting protein–peptide complex structures. This was because the original version of AutoDock Vina was too slow to run a large-scale peptide screening study. The computational time of AutoDock Vina mainly depends on two parameters: the *exhaustiveness* value and the number of steps (*N*) of the binding mode sampling algorithm (i.e. the iterated local search [ILS] global optimizer (44)). The *exhaustiveness* value determines the number of independent runs, in which different binding locations and orientations of a peptide on a target protein can be used as the starting points for the ILS global optimizer. The number of searching steps, *N*, is calculated as 210 × (50 + *m* + 10*n*)/2, where *m* is the number of movable atoms of a ligand and *n* is the number of degrees of freedom, including six degrees of translational and rotational freedom and the number of torsional angles (i.e. rotatable bonds) in the ligand. Interestingly, we discovered that increasing the *exhaustiveness* value (i.e. allowing for more independent runs) and decreasing the number of searching steps *N* in each independent run significantly accelerates AutoDock Vina without compromising its accuracy in predicting protein–peptide complex structures.

Specifically, in Vina_pep, the *exhaustiveness* value was increased from the default value of 8 to 64, and the number of searching steps *N* was decreased from the default value of 210 × (50 + *m* + 10*n*)/2 to (*m* + 10*n*). Both AutoDock Vina and Vina_pep were evaluated on the PepPro dataset. For each docking, the protein structure was extracted from the crystal structure of the protein–peptide complex and treated as a rigid body during docking. The peptide structure was also taken from the crystal complex structure, but the sidechain conformations were rebuilt using the Rotamers tool implemented in the UCSF Chimera (33). Peptide sidechains were treated as being fully flexible, and backbone atoms were treated as being rigid. The center of the searching box was set at the geometry center of the co-bound peptide in the crystal complex structure. The box size in each dimension was set at 1.5 times the size of the co-bound peptide structure in the corresponding dimension. The *exhaustiveness* value was set to 8 and 64 for AutoDock Vina and Vina_pep, respectively. Other parameters were set to default.

### Peptide ranking

After docking, a hybrid scoring function, PepProScore, was employed to rank the peptides in the library. PepProScore is defined as the sum of Vina_Score and *w* × PC_Score, where Vina_Score is the binding score of a predicted binding mode calculated with the scoring function implemented in Vina_pep (same as the scoring function in AutoDock Vina). PC_Score is the similarity score between a predicted protein–peptide interacting interface and the interface of the corresponding sequence fragment in the monomeric protein (22). The values of PC_Score range from 0 to 1, where 0 means no similarity and 1 indicates identical interfaces. The contributions of these two scores, the binding score and the similarity score, are balanced by the weight *w*, which is set to −9 based on our previous studies on the protein–peptide complex structure prediction (21).

For each peptide in the library, PepProScore was calculated for the top 10 binding modes generated by Vina_pep. The binding mode with the best PepProScore (i.e. the most negative score) was kept for each peptide in the library and then ranked according to PepProScore.

### Peptide candidate selection

Several automated filters were applied to the peptide ranking list to improve the hit rate in this study. First, peptides with many exposed hydrophobic residues in their predicted binding modes were discarded because our scoring function did not explicitly include the contribution from solvation. Specifically, peptides were removed from the ranking list if more than half of their residues were hydrophobic and if more than half of the hydrophobic residues (i.e. Ala, Val, Leu, Ile, Pro, Phe, Met, and Trp) were exposed in the predicted binding mode. A peptide residue was defined as an exposed residue if its relative buried surface area was >66.7%. The calculation was based on the predicted protein–peptide complex structure using the program Naccess V2.1.1 (30).

Second, because our peptide synthesis was usually problematic if a peptide contained more than three consecutive hydrophobic residues, such peptides were removed from the ranking list. Third, peptide sequences containing proline were also discarded because proline tends to destabilize the alpha-helix structure. It is noteworthy that these two filters can be applied to the peptide library before in silico screening to save computational time.

After applying these three filtering criteria to the protein target TEM-1, the first 10 peptides with a PC_score of ≥0.5 in the ranking list were selected for synthesis and assays.

### Synthesis of short peptides

All the peptides selected from in silico screening were synthesized in solid phase, using Sieber amide resin and standard Fmoc peptide chemistry with a Tetras multiple peptide synthesizer (purchased from Occam Design). Piperazine was used for Fmoc deprotection and HBTU/DIEA for coupling at each cycle. The permanent protection groups chosen for the amino acid sidechains were tBu (Tyr, Ser, and Thr), OtBu (Glu and Asp), Trt (Cys, His, Glu, and Asn), Boc (Trp and Lys), and Pbf (Arg). Capping was performed at the end of each cycle. After final Fmoc deprotection of the N-terminal amine, the peptides were cleaved from the resin and the side chain protection groups were removed in a single reaction with TFA, TA, phenol, water, EDT, and TIS (87.5:2.5:2.5:2.5:2.5:2.5) for 2 h at room temperature (25 °C). Precipitation and multiple washings with diethyl ether produced the final crudes. The final products were characterized and, when necessary, purified using MS-assisted HPLC (Beckmann Coulter Gold System HPLC coupled to a Thermofisher Ion trap Mass spec). Reverse phase C18 and C4 columns (from Waters and Thermo Fisher) were used for analyses and purification of the peptides.

### Protein expression and purification

The *E. coli* TEM-1 β-lactamase was a gift from Niels Geijsen (Addgene plasmid #62729; http://n2t.net/addgene:62729; RRID:Addgene_62729). The recombinant protein was expressed in BL21 (DE3) cells. The expression was induced with 1 mM isopropyl β-D-1-thiogalacto-pyranoside at an optical density of 600 nm of 1.0. The cells were harvested after 24 h of incubation at 4 °C, resuspended in lysis buffer (30 mM Tris, 500 mM NaCl, 1.25% glycerol, 2 mM BME, and pH 7.2), and lysed by sonication at an amplitude of 50%, 5 s on and 15 s off on ice for 5 min (Sonics VC505). The TEM-1 protein was then purified using His-tag affinity chromatography with a Nickel column. Fractions containing TEM-1 were further purified by size exclusion chromatography (HiLoad 16/60 Superdex 200pg, Cytiva). The final protein was buffer exchanged and stored in a buffer containing 30 mM Tris-HCl (pH 7.2), 1 mM MgCl_2_, 140 mM KCl, 10 mM NaCl, and 2 mM dithiothreitol.

### β-Lactamase activity assay

*K*_m_ of nitrocefin hydrolysis by TEM-1 was determined by mixing 5 nM of TEM-1 with 4, 5, 6, 7, 8, and 9 μM of nitrocefin (APExBIO), and monitoring the OD at 520 nm using a microplate reader (BioTek Synergy 2 SLFP Multimode Reader). Peptide screening was performed with 5 nM TEM-1 mixed with 100 μM of each peptide. The peptide was preincubated with TEM-1 at 37 °C for 30 min prior to mixing with nitrocefin. In the dose–response assessment, TEM-1 was preincubated with peptide TF7 at various concentrations at 37 °C for 30 min. Nitrocefin was then rapidly mixed with the TEM-1: peptide solution to measure the absorbance change at 520 nm. The final concentrations of TEM-1 and nitrocefin in the mixture were 5 nM and 10 μM, respectively. The final peptide concentrations were 100, 50, 25, 12.5, 6.25, 3.12, 1.56, 0.78, 0.39, and 0.19 μM. The *V*_max_ was calculated by fitting the linear range of the kinetic data, and the *IC50* was calculated by fitting the DoseResp function in Origin 7.0. The inhibition constant, *K*_i_, which denotes the concentration of the inhibitor required to achieve half-maximal inhibition of the enzyme in the presence of a substrate, was determined by $K_{i}=(IC_{50}-E/2)/(S/K_{m}+1)$ (45). In this equation, *E* is the enzyme concentration, *S* is the substrate concentration, *K*_m_ is the Michaelis constant, and IC_50_ is the concentration of the inhibitor that reduces enzyme activity by 50%.

To evaluate the peptide inhibition mode, a Lineweaver–Burk double reciprocal plot was obtained. Briefly, 4 nM TEM-1 was preincubated with the peptide at various concentrations (0.9, 1.8, 3.6, 5.4, and 7.2 μM) at 37 °C for 30 min. Subsequently, different concentrations (4, 5, 6, 7, 8, and 9 μM) of nitrocefin were rapidly mixed with the TEM-1 and peptide solution to measure the absorbance at 520 nm.

### MST assay

To fluorescently label TEM-1 and its mutant proteins, 200 nM protein with the His-tag was incubated with 100 nM His-tag labeling dye (His-Tag labeling kit Red-tris-NTA 2nd generation, Monolith, NanoTemper) at room temperature for 30 min. After incubation, the sample was centrifuged for 10 min at 4 °C at 15,000*×g*. The labeled TEM-1 protein (50 nM) was then incubated with a serial dilution of peptide ranging from 450 to 0.027 µM for 30 min at 37 °C before being loaded onto a NanoTemper Monolith. The data were analyzed using MO.Affinity Analysis (NanoTemper). Three independent replicates were carried out to measure the dissociation constant *K*_d_, which represents the equilibrium constant for the dissociation of the inhibitor from its binding site on the enzyme. For M270R, which exhibited a biphasic binding curve, the *K*_d_ value was determined by excluding data points for the low-affinity binding mode during the analysis of the high-affinity binding mode, and vice versa (46).

## Data Availability

The MDockPeP2_VS program and data presented in the manuscript are available at https://zougrouptoolkit.missouri.edu/mdockpep2_vs/download.html.
